# *Coxiella burnetii* infections in mice: Immunological responses to contemporary genotypes found in the US

**DOI:** 10.1080/21505594.2021.1975527

**Published:** 2021-09-13

**Authors:** Rachael A. Priestley, Cody B. Smith, Halie K. Miller, Gilbert J. Kersh

**Affiliations:** Rickettsial Zoonoses Branch, Division of Vector-Borne Diseases, Centers for Disease Control and Prevention, Atlanta, United States

**Keywords:** *Coxiella burnetii*, Q fever, mouse model, immunology, genotypes, isolates, aerosol infection

## Abstract

*Coxiella burnetii* is an obligate intracellular bacterium that causes the human disease Q fever, which can manifest as an acute flu-like illness or a long-term chronic illness, such as endocarditis. Three genotypes (ST8, ST16, and ST20) of *Coxiella burnetii* are commonly found in the contemporary US and are associated with specific animal hosts. Although all three genotypes have been isolated from humans with Q fever, studies comparing virulence between *C. burnetii* sequence types have been rare. Here, groups of mice were infected via aerosol inoculation with isolates derived from cow’s milk, environmental, animal, and human samples. Mice were monitored for weight loss and blood samples were takenweekly. Animals were euthanized at 2- and 12-weeks post-infection, and bacterial burden was determined for tissues by real-time PCR. The levels of anti-Coxiella antibodies and selected inflammatory cytokines were determined for serum samples. Weight loss and splenomegaly were observed in mice infected with ST20 and ST16 isolates but were absent in the mice infected with ST8 isolates. Bacterial concentrations in the tissues were lower in the ST8 isolates at 2 weeks post-infection relative to all other isolates. ST16 and ST20 isolates induced robust antibody and cytokine responses, while ST8 isolates produced significantly lower anti-*C. burnetii* titers early in the infection but saw increased titers in some animals several weeks post-infection. The data suggest that the ST8 isolates are less virulent in this mouse model, as they produce less robust antibody responses that are slow to develop, relative to the ST16 and ST20 isolates.

## Introduction

*Coxiella burnetii* is a zoonotic, obligate intracellular bacteria that causes the disease Q fever. *C. burnetii* is found throughout the animal world, with the highest risk of human disease coming from exposure to infected animal birth products, milk, feces, or the dust contaminated by these animal products [1]. Acute infections often present as a non-descript flu-like illness, with severe cases developing into atypical pneumonia or possibly hepatitis [2]. Approximately 1–5% of acute Q fever infections can become latent and develop into chronic Q fever months or years later. Chronic Q fever typically manifests as endocarditis but can also present as vascular infections, hepatitis, osteomyelitis, or psoas abscesses [3]. Q fever is endemic worldwide, with the exception of New Zealand, and the most common human exposure risk in the US is generally associated with livestock husbandry [4,5].

Several genotyping schemes have been used to differentiate *C. burnetii* strains, with Multispacer Sequence Typing (MST) being used to define over 70 unique sequence types [6]. A rapid PCR-based method of genotyping was developed that uses single nucleotide polymorphisms (SNPs) to identify the sequence types defined by MST genotyping [7]. Using this method, we have determined that there are primarily three sequence types (ST) of *C. burnetii* currently circulating in the United States and two of these sequence types have been linked to specific animal hosts [8]. ST20 isolates are associated with cattle, are commonly found in commercial dairy products, but are not often implicated in human infections [9]. ST8 isolates are typically associated with goats in the U.S., have been linked to recent human outbreaks, and have been associated with chronic Q fever infections [8,10]. ST16 isolates include Nine Mile (9Mi), the type strain of *C. burnetii* that was isolated from a tick in 1935. ST16 isolates lack a strong linkage to specific hosts and have been isolated from infected persons in the past. ST8 isolates also contain an extra-chromosomal plasmid that differs from the plasmid found in ST16 and ST20 isolates and has been implicated as a possible factor of disease presentation [6,11].

It is thought that the most common route of infection of Q fever is through inhalation, although infection through ingestion is a possibility [12]. Infections of mice and guinea pigs have been previously studied using intraperitoneal injection and inhalation of contaminated aerosols with strains isolated 40–80 years ago with extensive lab passage histories [13–15]. To define immunological and pathological differences among sequence types currently circulating in the US, we have evaluated the *in vivo* growth, antibody responses, cytokine responses, and clinical signs of infection in immunocompetent mice infected with *C. burnetii* by aerosol inhalation. C57Bl/6 mice were infected with three isolates of each sequence type and followed over a period of 12 weeks. Serum samples were taken regularly and used to determine antibody response curves and inflammatory cytokine levels, and tissues were collected at the end of the study to assess bacterial burden in multiple organs. In addition, groups of mice were infected with two strains closely related to ST8 isolates, but with origins in Australia and Central Asia.

## Materials and methods

### Propagation of isolates

Three isolates from each US sequence type were chosen for animal infection. ST16 isolate Nine Mile (9Mi) Phase I, the type strain of *C. burnetii*, was used for comparison to all other isolates [16]. Two other isolates that are closely related to 9Mi and genotype in a group that includes ST16 and ST26 were HPF-GA1, a strain isolated from a human chronic infection [17], and ES-VA1, which was isolated from an environmental sample [8]. The ST20 isolates were CM-CA1 and CM-SC1, both isolated from commercially available unpasteurized cow milk [18], and HA-WI2, which was derived from a human chronic infection. The presence of the QpH1 plasmid in the ST16/26 and ST20 isolates was confirmed by PCR [8]. The ST8 isolates used were GP-CO1, from an infected goat placenta [19], PB-CA2, isolated from a human chronic infection, and ES-CA1, from an environmental sample [8]. The presence of the QpRS plasmid in the ST8 isolates was confirmed by PCR [8]. Apart from 9Mi Phase I, all US strains used were isolated from samples collected in the US within the last 15 years (Table 1). Additionally, two non-American isolates were chosen to compare to the above-mentioned isolates. AuQ17 was gifted by Dr. Gemma Vincent of the Australian Rickettsial Reference Laboratory (Victoria, Australia). This isolate was derived from a serum sample obtained from an acute Q fever patient in 2011 in Australia and although it does not match any of the sequence types identified by MST, it is closely related to the ST1-7 group and has the QpRS plasmid-like ST8 isolates [20]. RT-Schperling was gifted to us by Dr. Dimitrios Frangoulidis of the Bundeswehr Institute for Microbiology (Munich, Germany). RT-Schperling was isolated from a human blood sample in Krygyzstan in 1955 and has sequence-type ST2 [6]. Although it is closely related to the Australian isolate it does not contain the QpRS plasmid but was confirmed to contain the QpDV plasmid. Each *Coxiella* isolate was grown in the cultured rabbit kidney cell-line RK-13 until the cells were heavily infected and beginning to lyse. *C. burnetii* was then purified by digitonin purification [21] and quantitated by *com1* real-time quantitative PCR [22]. SNP genotyping assays were performed on the purified isolates to verify that no cross-contamination occurred during growth [7]. To ensure that the isolates contained full-length lipopolysaccharide (LPS), LPS structures were extracted from 2 × 10^9^ genome equivalents of purified *C. burnetii* using the hot phenol-water extraction method, analyzed by SDS-PAGE, and observed by silver staining as previously described [23]. Purified isolates were stored in sucrose phosphate glutamate (SPG) buffer and frozen at −80 C until use.

**Table 1.** *C. burnetii* isolates used in this study

Isolate	Sequence Type	Year Isolated	Plasmid	Source	Reference
9Mi I	ST16/26	1935	QpH1	Tick in Montana	16
HPF-GA1	ST16/26	2016	QpH1	Human psoas abscess	17
ES-VA1	ST16/26	2008	QpH1	Environmental sample	8
CM-CA1	ST20	2007	QpH1	Raw cow milk	18
CM-SC1	ST20	2007	QpH1	Raw cow milk	18
HA-WI2	ST20	2016	QpH1	Human aorta	current
GP-CO1	ST8	2008	QpRS	Goat placenta	19
ES-CA1	ST8	2008	QpRS	Environmental sample	8
PB-CA2	ST8	2016	QpRS	Human peritoneal biopsy	current
AuQ17	ST1-7/30	2011	QpRS	Acute Human sera	20
RT-Schperling	ST7	1955	QpDV	Human blood, Krygyzstan	6

### Infection of mice

For each isolate, eight male C57Bl/6 mice, 6–8 weeks of age, were placed into the Biaera aerosol chamber (Biaera Technologies, Hagerstown, MD) and 10^8^ organisms/ml of *Coxiella* from frozen stocks was aerosolized using a collision nebulizer for 10 minutes, allowing an infectious dose of approximately 6.8 × 10^4^ organisms per mouse [24]. Animals exposed to sterile PBS served as the negative control group. 10 ml of sterile PBS was added to an impinger that sampled air flowing through the Biaera chamber during exposure. After exposure, 200 µl of the impinger sample was extracted and quantitated by *com1* PCR to determine the actual infectious dose (Table 2). An additional 1 ml of the sample was placed onto RK13 cells and allowed to grow to verify the viability of each isolate.

**Table 2.** Dose of *C. burnetii* inhaled

Isolate	Target infectious dose	Actual infectious dose
9Mi	6.8x10^4^	5.8x10^4^
HPF-GA1	6.8x10^4^	6.4x10^4^
ES-VA1	6.8x10^4^	9.8x10^4^
CM-SC1	6.8x10^4^	7.4x10^4^
CM-CA1	6.8x10^4^	6.1x10^4^
HA-WI2	6.8x10^4^	1.2x10^4^
GP-CO1	6.8x10^4^	5.9x10^4^
ES-CA1	6.8x10^4^	2.3x10^5^
PB-CA2	6.8x10^4^	6.2x10^4^
AuQ17	6.8x10^4^	8.8x10^4^
RT-Schperling	6.8x10^4^	2.2x10^5^

Throughout the study, mice were housed in an Tecniplast IsoCage Biocontainment system (Tecniplast, Exton, PA) in an ABSL3 facility and provided food and water *ad libitum*. Mice were monitored for clinical symptoms, such as ruffled fur, huddling, and weight loss daily during the first two weeks post-infection (pi), then twice a week for the duration of the study. Blood samples were taken via the submandibular vein on days 1, 4, 7, 10, and 14 pi, then weekly for an additional 10 weeks. Three animals from each infected group were euthanized on day 14 and necropsies were performed. The remaining five animals of each infection group were euthanized and necropsied 12 weeks pi. One animal from the GP-CO1 group was euthanized and necropsied at 34 days pi due to pathology unrelated to Q fever. Tissues harvested during necropsy were spleen, liver, lungs, heart, testes, bone marrow, and adipose. After necropsy spleens were weighed to assess splenomegaly.

### PCR analysis

Tissues were homogenized in 300 µl sterile PBS in tubes containing 3.0 mm triple-pure zirconium beads in a BeadBug 6 microtube homogenizer (Benchmark Scientific, Edison, NJ). The BeadBug homogenization protocol consisted of 5 cycles of 7.0 m/s for 30 seconds, with a 30 second pause between each cycle. 100 µl of the tissue homogenates were then extracted using the QIAamp DNA Mini Kit (Qiagen Inc., Germantown, MD). The initial lysis step using ATL tissue lysis buffer and proteinase K was performed with an overnight 56°C incubation. The manufacturer’s protocol was followed for the remaining steps. The tissue DNA samples were then analyzed for the presence of *C. burnetii* DNA by real-time quantitative PCR. PCR was performed using a single-copy *com1* assay to target *Coxiella* DNA [25], and a commercial murine *β-actin* (Actb) endogenous control assay to target mouse DNA (Applied Biosystems, Waltham, MA). All PCR was performed on an ABI 7500 Fast instrument (Applied Biosystems, Waltham, MA) and analyzed using the manufacturer provided SDS software. Cycle threshold (C_t_) data for the *C. burnetii com1* gene were normalized to murine Actb and the normalized cycle threshold values (ΔC_t_) were transformed using 2^−ΔCt^/10^−6^ and reported as arbitrary quantity units as described previously [12]. Tissues were also tested using a multi-copy *IS1111* assay in order to detect lower amounts of *C. burnetii* DNA [25].

### Immunological analysis

Blood samples were taken from each mouse by venipuncture of the submandibular vein using a Goldenrod Animal Lancet (Fisher Scientific, Philadelphia, PA) and collected using Sarstedt Microvette 100 Z serum collection tubes (Sarstedt, Germany). 50 μl of blood was taken from each animal on days 1, 4, 7, and 10 pi. 100 μl of blood was taken weekly from day 14 to week 12 pi. Collection tubes were centrifuged at 10,000 × g for 5 minutes and serum was transferred to Sarstedt tubes and frozen at −80°C. Serum samples were gamma-irradiated at 2 × 10^6^ rads to inactivate any remaining *C. burnetii* present. An in-house immunofluorescence assay (IFA) for IgG and IgM antibodies was run as previously described [24] on all samples to determine the antibody titer at each time point. Geometric mean titers were calculated for each infected group to graph the trend of antibody levels over time [26].

Serum samples were prepared for cytokine analysis by pooling 5 μl of serum from each animal in an infected group at days 4, 7, and 10 pi. The pools were then run in duplicate on a multi-plex cytokine assay kit (Bio-Rad, Hercules, CA). Analysis was performed using the Bio-Plex platform on a MagPix instrument (Bio-Rad, Hercules, CA). The observed concentration of each cytokine was calculated for fold change of the isolate relative to PBS at each time point. Changes greater than 2-fold were considered significant.

### Statistical analysis

Weight loss was analyzed for statistical significance via Student’s *t*-test. Splenomegaly data were analyzed for statistical significance by two-way ANOVA with Dunnett’s correction for multiple comparisons. Data analysis was performed using the GraphPad Prism 8 software (GraphPad, San Diego, CA). *P* < 0.05 was deemed significant in all analyses. Confidence intervals for fold change in Figure 6 were calculated according to the method of Fieller [27].

**Figure 1.**
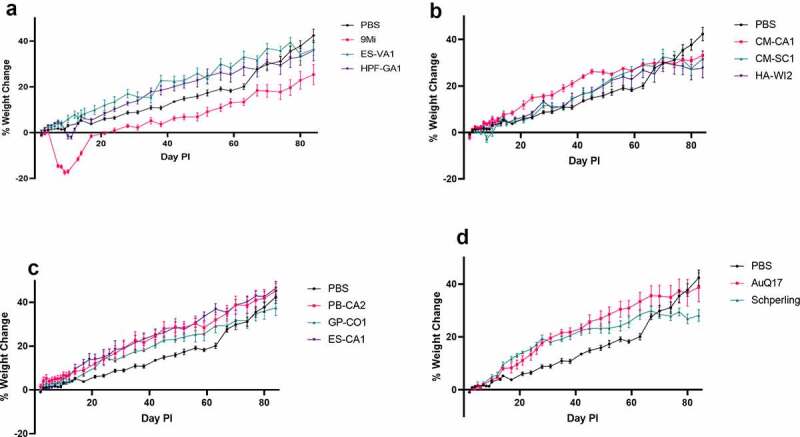
**Weight change in mice after infection with isolates of *C. burnetii***. Mice were infected with *C. burnetii* isolates via inhalation and weight was monitored for 12 weeks (84 days). Weights are expressed as percent change compared to weight at day 1 pi. Mice are grouped by sequence type; (a) ST16/26, (b) ST20, (c) ST8, (d) AuQ17 and RT-Schperling. The mean percent change ± SEM for each group of mice infected with an isolate is displayed at each time point

**Figure 2.**
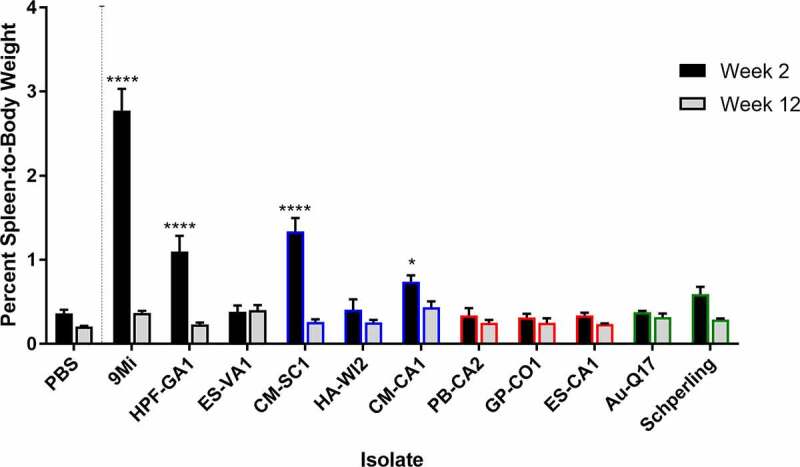
**Splenomegaly in mice after infection with isolates of *C. burnetii***. Mice were infected with *C. burnetii* isolates via inhalation and spleen weight was measured at 2 weeks (black) and 12 weeks (gray) pi. Spleen weights were calculated as the percentage of body weight for each mouse. The figure shows the mean ± SEM for each group of mice infected with an isolate. Statistical significance was determined by a two-way ANOVA with Dunnett’s correction for multiple comparisons relative to PBS at the corresponding time point, *p < 0.05, ****p < 0.0001. Sequence types are grouped by color: ST16/26 (black), ST20 (blue), ST8 (red), and ST1-7/30 (green)

**Figure 3.**
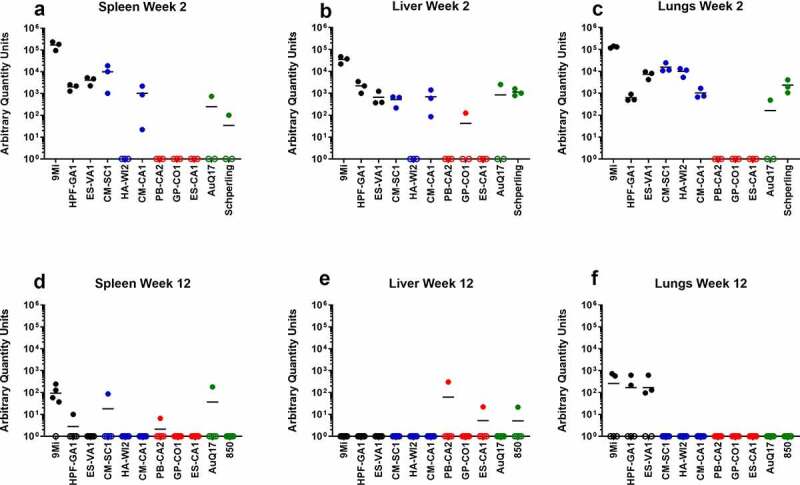
**Detection of *C. burnetii* by PCR in organs of mice after infection with isolates of *C. burnetii***. Mice were infected with *C. burnetii* isolates via inhalation and spleens (a, d), livers (b, e), and lungs (c, f) were harvested at 2 weeks (a-c) and 12 weeks (d-f) pi. DNA was prepared and quantitative PCR was performed using an assay to detect the *C. burnetii com1* gene and normalized to a murine *β-actin* endogenous control PCR assay. The results are reported as arbitrary quantity units, and each point represents an individual mouse. Sequence types are grouped by color: ST16/26 (black), ST20 (blue), ST8 (red), and ST1-7/30 (green)

**Figure 4.**
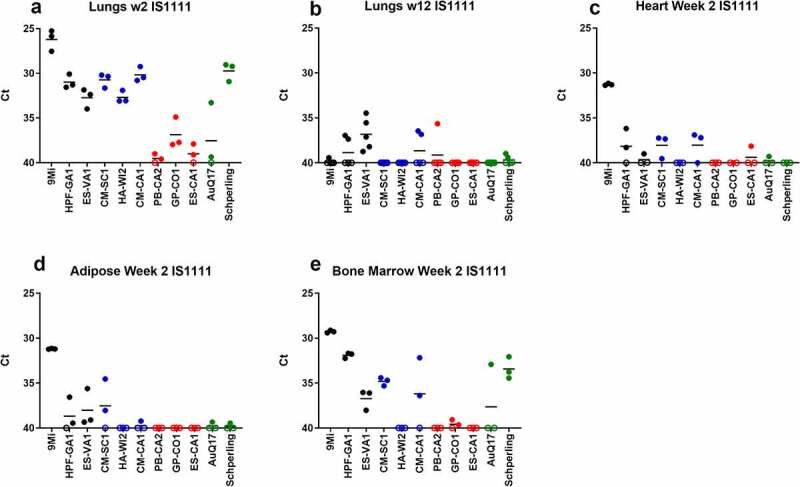
**Detection of *C. burnetii* by PCR in organs of mice after infection with isolates of *C. burnetii***. Mice were infected with *C. burnetii* isolates via inhalation and lungs (a-b), hearts (c), adipose tissue (d), and bone marrow (e) were harvested at 2 weeks (a, c) and 12 weeks (d-f) pi. DNA was prepared and quantitative PCR was performed using an assay to detect the *C. burnetii IS1111* gene. The *IS1111* target is present in the *C. burnetii* genome in multiple copies making this assay more sensitive than detection of the single copy *com1* gene. Variable copies of *IS1111* among *C. burnetii* isolates makes the results between isolates not quantitatively comparable. Results are reported as Ct values, and each point represents an individual mouse. Sequence types are grouped by color: ST16/26 (black), ST20 (blue), ST8 (red), and ST1-7/30 (green)

**Figure 5.**
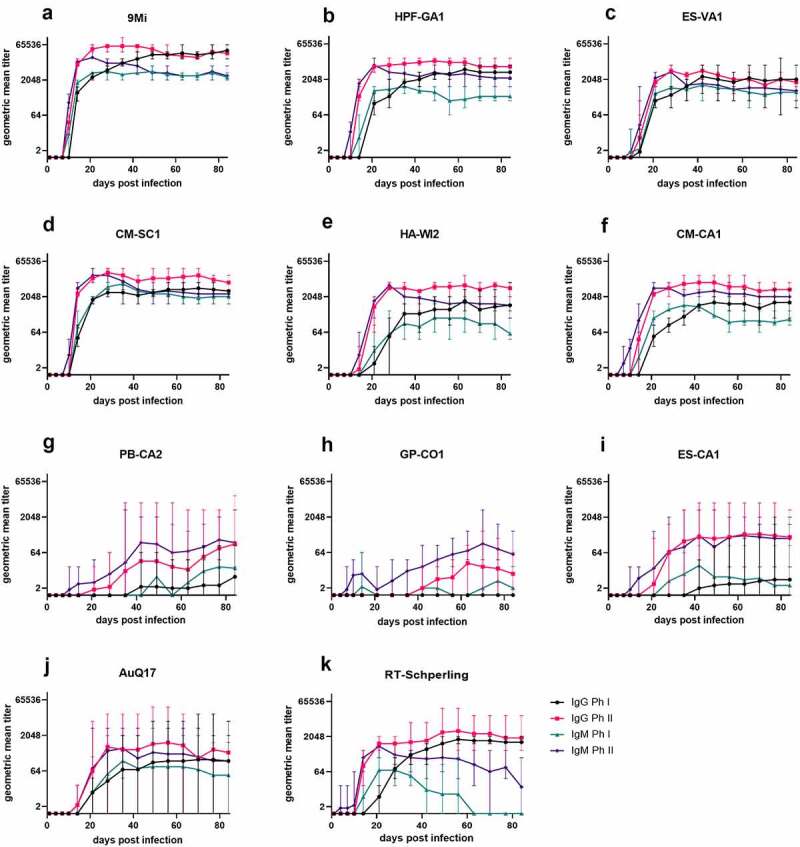
**Antibody responses in mice after infection with isolates of *C. burnetii***. Mice were infected with *C. burnetii* isolates via inhalation and serum antibody levels to *C. burnetii* phase I and phase II antigens were measured for 12 weeks (84 days). The geometric mean titer ± range is displayed for each group of mice infected with the indicated isolates. Antibodies IgM phase II (purple), IgM phase I (green), IgG phase II (red), and IgG phase I (black) were detected using an in-house IFA

**Figure 6.**
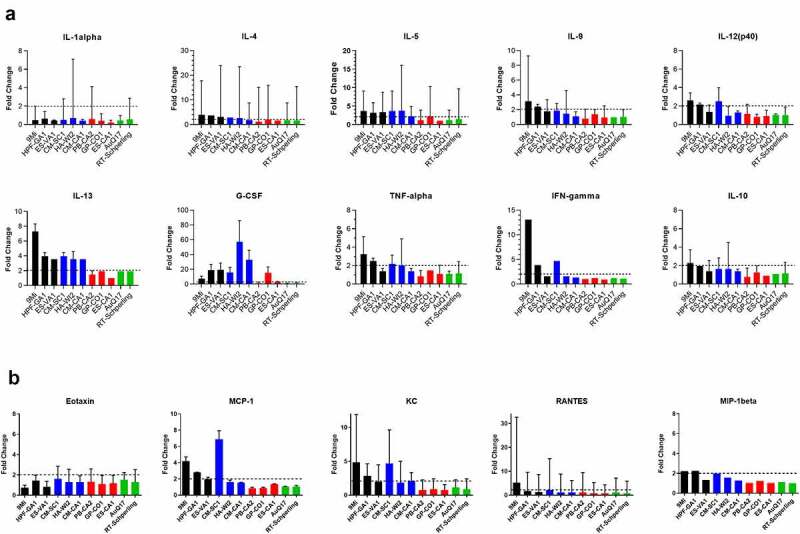
**Cytokine responses in mice after infection with isolates of *C. burnetii***. Mice were infected with *C. burnetii* isolates via inhalation and serum from groups of mice infected with the same isolate were pooled for cytokine analysis using a magnetic bead-based Bio-Plex assay on a MagPix instrument. Fold change in (a) cytokine and (b) chemokine levels at day 10 pi relative to PBS infected mice are displayed. Changes larger than 2-fold (dotted line) were considered significant. Error bars represent 95% confidence intervals for fold change. Confidence intervals for IFN-gamma and MIP-1 beta were not possible to calculate using Fieller’s method. Sequence types are grouped by color: ST16/26 (black), ST20 (blue), ST8 (red), and ST1-7/30 (green)

## Results

C57BL/6 mice were infected with *C. burnetii* isolates by inhalation of aerosolized bacteria. An infectious dose of 6.8 × 10^4^ organisms was targeted. The actual doses were calculated by performing quantitative PCR on a sample from an impinger and ranged from 1.2 × 10^4^ to 2.3 × 10^5^ organisms per animal (Table 2). Animals were weighed at least twice weekly after infection, and weights were converted to percent weight change relative to day 1 pi (Figure 1). For the animals infected with ST16/26 isolates (Figure 1a), 9Mi caused the most severe weight loss, with animals losing nearly 20% of weight by day 10 pi. HPF-GA1 also caused weight loss by day 10 pi of up to 5%, while animals infected with ES-VA1 showed no weight loss compared to PBS controls. The weight loss caused by 9Mi and HPF-GA1 at day 10 pi was considered statistically significant (p < 0.0001 and p = 0.0063, respectively) relative to the PBS controls. Of the ST20 isolates (Figure 1b), only CM-SC1 caused any statistically significant weight loss (p = 0.0006), with animals losing up to 5% by day 10 pi. Interestingly, animals infected with all three ST20 isolates showed a plateau of weight that appeared around week 11 pi and was statistically significant (CM-CA1, p = 0.03; CM-SC1, p = 0.04; HA-WI2, p = 0.02) relative to the PBS controls by week 12 pi. Animals infected with the three ST8 isolates (Figure 1c) showed no signs of weight loss, and their weight gain over time was unremarkable when compared to the PBS controls. The AuQ17 and RT-Schperling isolates (Figure 1d) also caused no significant weight loss early in the infection, although RT-Schperling did cause a weight plateau like that seen in the ST20 isolates that was statistically significant (p = 0.0042) by week 12 pi.

Upon necropsy, spleens were weighed to assess splenomegaly before further processing. Spleen weights are reported as a percentage of body weight (Figure 2). Mice infected with 9Mi showed the most significant splenomegaly (2.8%, p < 0.0001) at two weeks pi, and splenomegaly for animals necropsied at 12 weeks pi was reduced compared to week 2 and no longer significant (0.37% versus 0.21% for PBS controls, p = 0.3927). The ST16/26 isolate HPF-GA1 also caused significant (1.1%, p < 0.0001) splenomegaly at 2 weeks pi, but there were no signs of splenomegaly at 12 weeks pi. The final ST16/26 isolate, ES-VA1 did not cause splenomegaly at 2 weeks nor at 12 weeks pi. Of the ST20 isolates, only one isolate derived from cow’s milk, CM-CA1, caused splenomegaly at 2 weeks pi (0.74%, p = 0.01). HA-WI2 did not appear to cause any inflammation of the spleen at either time point; however, this could be caused by its reduced inoculating dose during infection (Table 2). None of the ST8 isolates nor the AuQ17 and RT-Schperling caused any splenomegaly at either time point when compared to the PBS controls.

All tissues processed for DNA extraction were tested by quantitative PCR using an assay to detect the *C. burnetii com1* gene and normalized to a murine *β-actin* endogenous control PCR assay, and results are reported as arbitrary quantity units (Figure 3). *C. burnetii* DNA was consistently detected by *com1* PCR in the spleen, liver, and lung of animals infected with the ST16/26 and ST20 isolates, while it was below the limit of detection in these tissues from animals infected with ST8 isolates (Figure 3a-c). Only one animal infected with AuQ17 had detectable *C. burnetii* DNA at week 2 pi, and three animals infected with RT-Schperling had detectable *C. burnetii* DNA in liver and lung at week 2 pi. Infection of animals with 9Mi resulted in the highest bacterial load in the spleen, liver, and lungs at 2 weeks pi. 9Mi also appeared to persist in the spleens of infected animals throughout the duration of the study, with 4 of the 5 animals having detectable levels of DNA at 12 weeks pi (Figure 3d). In the liver at 12 weeks, only a few animals had detectable *C. burnetii* DNA. These were two mice infected with ST8 isolates, and one infected with RT-Schperling (Figure 3e). All three of the ST16/26 isolates had detectable levels of *C. burnetii* DNA in the lungs of some animals at 12 weeks pi (figure 3f). This persistence was not seen consistently in the lung tissues of animals infected with isolates from the other sequence types. Tissue samples were also tested using an *IS1111* assay in order to detect levels of *Coxiella* DNA that were below the limit of detection of the *com1* assay. *IS1111* is a transposable element that is present in *C. burnetii* DNA in varying copy numbers depending on the isolate being tested. The increased copy numbers of *IS1111* allow the assay to detect lower concentrations of *C. burnetii* DNA than the *com1* assay, however, the *IS1111* assay cannot be used for quantitation as each isolate has a different number of *IS1111* copies. Typically, ST16/26 and ST20 isolates contain 20–30 copies of *IS1111*, while ST8 and ST1-7/30 isolates can contain more than 50 copies of the gene sequence. The *IS1111* results are reported by C_t_ value only (Figure 4). This more sensitive assay demonstrated that there was detectable *C. burnetii* DNA in the lungs of most animals infected with ST8 isolates, AuQ17, and RT-Schperling at 2 weeks pi (Figure 4a), but by 12 weeks most of these mice were negative by PCR. Low levels of *C. burnetii* DNA were detectable in the heart, adipose, and bone marrow samples of animals infected with the ST16/26 and ST20 isolates, while DNA levels were at or below the limit of detection in these tissues for all animals infected with ST8 isolates (Figure 4c-e). The testes do not appear to be a consistent reservoir of *Coxiella* in C57Bl/6 mice, with only the three 9Mi-infected animals having consistent levels of DNA detectable in the testes at week 2 pi. One of each group infected with CM-SC1 and HPF-GA1 had PCR positive testes at week 2 pi. The testes of all animals were PCR negative by 12 weeks pi (data not shown).

Serum samples were tested by an in-house IFA assay that detects IgG and IgM antibodies against 9Mi Phase I and Phase II antigens. Titers for each infected group were transformed into geometric mean titers and are reported as the reciprocal GMT (Figure 5). Titers of animals infected with ST16/26 and ST20 isolates followed similar trends, with anti-Ph II antibodies developing first starting between days 7 and 10 pi, followed by anti-Ph I antibodies developing beginning between days 10 and 14 pi. IgM antibodies peaked between weeks 3 and 4 pi and were somewhat reduced by week 12, while IgG antibodies remained near their peak for the duration of the study. Animals infected with 9Mi had the highest titers overall, with a peak titer of 1:65,536 by week 4 pi. While the remaining ST16/26 and ST20 isolates did not cause titers as high as 9Mi, their peak titers were all above 1:4096 within the same time frame (Figure 5). The titers of animals infected with ST8 isolates were highly variable within each infected group. Most of the animals infected with these isolates had a low-titer response, with only 40% of the ST8-infected animals developing a Ph II titer greater than 1:128 several weeks into the study. Only one animal in the GP-CO1-infected group developed a titer ≥1:128 approximately 7 weeks pi. Two animals in the PB-CA2 cohort developed titers ≥1:128 starting 4 weeks pi, and three animals in the ES-CA1-infected group began developing titers ≥1:128 around 3 weeks pi. While anti-Ph II antibodies of ST8-infected animals varied greatly, all anti-Ph I antibodies from these animals remained below 1:128 for the duration of the study (Figure 5). Animals infected with AuQ17 had varied serological results similar to those seen within the ST8-infected groups, however, animals that developed titers ≥1:128 developed both anti-Ph I and anti-Ph II antibodies between 2- and 3-weeks pi. The animal group infected with RT-Schperling had antibody responses that more closely followed the trends of the ST16/26 and ST20 infected mice, however the overall titers were much lower, peaking below 1:4096 (Figure 5).

Due to limited volume of the serum samples, cytokine and chemokine analysis was performed on pools of serum from each infected group at days 4, 7, and 10 pi (Supplemental Table 1). The samples were evaluated using a commercially available multi-plex cytokine assay. Cytokine levels at day 10 were graphed based on the fold-change of each infected group relative to PBS-infected mice (Figure 6), with any change greater than 2-fold being considered significant. Some cytokines had slightly higher induction on day 7, but day 10 best demonstrates the pattern of induction among the different isolates. ST16/26 infection resulted in a greater than 2-fold induction for most of the cytokines. 9Mi resulted in induction for 9/10 cytokines and 4/6 chemokines, and HPF-GA1 resulted in 2-fold induction in 8/10 cytokines and 3/6 chemokines. ST20 isolates also caused cytokine induction with CM-SC1 resulting in induction of 8/10 cytokines and 3/6 cytokines, and HA-WI2 infection inducing 5/10 cytokines but 0/6 chemokines. Infection with ST8 isolates did not result in induction of many cytokines, with PB-CA2, GP-CO1, and ES-CA1 infection inducing 0/10, 3/10, and 1/10 cytokines, respectively. No chemokines were induced by the three ST8 isolates. The AuQ17 and RT-Schperling isolates behaved similarly to the ST8 isolates, with no cytokines or chemokines induced by either of these isolates.

The most robust induction was seen with G-CSF, with 7/11 isolates causing induction and HA-WI2 inducing G-CSF 57-fold compared to PBS-injected mice. IL-13 was also broadly induced with all six of the ST16/26 and ST20 isolates causing greater than 2-fold induction. IL-1alpha was the only cytokine tested that was not induced by any of the isolates. IL-10 was only induced by 9Mi and CM-SC1, and for these isolates the induction was only slightly above 2-fold. Although most of the cytokines and chemokines were highest at day 10, Eotaxin was the exception, with most isolates inducing greater than 2-fold above PBS-infected mice on day 4, with levels declining by day 10.

An unexpected finding was the development of hemothoraxes in some animals necropsied at 2 weeks pi. Two out of three animals in the HPF-GA1 infected group, all three of the mice in the CM-CA1 infected group, and 2 of 3 animals in the CM-SC1 infected group had hemothoraxes that were PCR positive for *C. burnetii* DNA. No PCR-positive hemothoraxes were found at 12 weeks pi, and none of the animals with ST8 or ST1-7 isolates were found to have PCR-positive hemothoraxes at 2 weeks pi.

## Discussion

**Figure 7.**
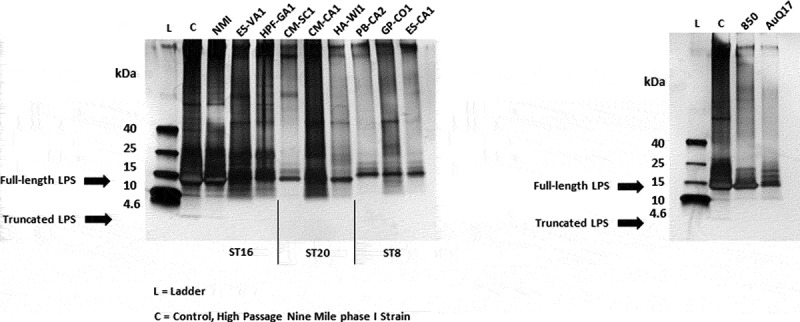
**Analysis of LPS from *C. burnetii* strains**. LPS was extracted from purified cell culture grown *C. burnetii*. Extracts were separated by sodium dodecyl sulfate-polyacrylamide gel electrophoresis and observed by silver staining. A protein ladder (L) and high-passage Nine Mile phase I control strain (C) are included. The upper (10–14 kDa) and lower (2.5–4 kDa) arrows indicate full-length and truncated LPS structures, respectively. LPS, lipopolysaccharide

Previous studies have examined the response of immunocompromised and immunocompetent mice and guinea pigs to *Coxiella burnetii* isolates delivered by intraperitoneal injection and/or inhalation [14,15,24,28]. These studies have generally found greater weight loss, splenomegaly, splenic bacterial load, and cytokine induction after infection with ST16 isolates compared to the infection with ST8 isolates. Using a different set of isolates and infection by inhalation of aerosols, the current results show a similar pattern – greater weight loss, splenomegaly, splenic bacterial load, cytokine induction, and antibody responses after infection with ST16/26 isolates compared to ST8. The current results also show that infections with ST20 isolates produce more significant infections than the ST8 isolates, with outcomes similar to the non-9Mi ST16 infections. We confirmed through visualization of the LPS structure for each isolate that the differences in virulence observed herein were not due to loss of full-length LPS (Figure 7).

The severity of mouse infections with the ST16/26 and ST20 isolates compared to the mild infections observed with ST8 isolates raise the prospect that plasmid sequences may play a role in virulence for *C. burnetii*. ST16/26 and ST20 isolates bear the QpH1 plasmid whereas ST8 isolates have QpRS. *C. burnetii* plasmids have been shown to encode type 4 secretion system (T4SS) substrates that are translocated into the host cell cytoplasm where they presumably subvert host cell functions to support the growth of *C. burnetii* [29]. Some genes are common to all *C. burnetii* plasmids, where others are plasmid specific. It is therefore possible that the absence of QpH1-specific genes on the QpRS plasmid contribute to the low virulence of isolates bearing QpRS. The presence of potential virulence genes on the QpH1 plasmid is supported by the low virulence of the AuQ17 and RT-Schperling isolates. AuQ17 is closely related to the ST8 isolates and also bears the QpRS plasmid, whereas RT-Schperling lies close to ST8 on the phylogenetic tree but has the QpDV plasmid [6,20]. It is therefore plausible that QpH1-specific genes may make isolates with this plasmid more virulent in the models that have been analyzed. However, it should be noted that the *C. burnetii* chromosome also differs between isolates that have QpH1 versus QpRS, and plasmid-specific virulence genes have not been identified.

Animal models infected with ST16/26 and ST20 isolates demonstrate greater bacterial growth, worse pathology, and more intense antibody and cytokine responses than animals infected with ST8 isolates. In the U.S., outbreaks of Q fever are often associated with ST8 isolates [19,30,31], and a number of ST8 isolates in the past 20 years have been derived from human chronic infections. The apparent association of ST8 isolates with human disease, but very low virulence in animal models could be explained by the species-specific reservoir adaptation of *C. burnetii* sequence types. ST20 *C. burnetii* are closely associated with dairy cattle, and infected dairy cows have been shown to shed *C. burnetii* abundantly in milk, but the cows seem to have limited pathology after infection [9]. In contrast, ST8 isolates are closely associated with goats in the U.S.[8] Recent Q fever outbreaks in the U.S. have been linked to goat farms [19,30,31]. These outbreaks involve widespread infection of goats, often with numerous associated abortions and stillbirths. High levels of *C. burnetii* in goat birth products results in substantial contamination of the local environment with *C. burnetii* [10]. Although these situations result in widespread environmental contamination, the number of human Q fever cases in these outbreaks is typically low. Recent U.S. outbreaks have resulted in 20–50 seroconversions with only about half of those seropositive reporting an illness clinically compatible with Q fever. This contrasts with the Netherlands outbreaks of 2007–2010, where a different sequence type (with the QpH1 plasmid) was spread in large amounts by goats and resulted in over 4,000 human Q fever cases [32]. Although occasional Q fever cases have been linked to cow’s milk, and ingestion is a potential mode of transmission of *C. burnetii*, this does not appear to be a common source for Q fever [12,33]. ST16/26 strains are not linked to a specific reservoir species but have been found in ticks and wild mammals. It is likely that limited contact with ST16/26 reservoirs makes this a relatively uncommon sequence type for human Q fever in the U.S.

One of the goals of this study was to examine potential tissue types that could serve as a reservoir for latent infections of *C. burnetii*. Bone marrow and adipose tissue have been previously described as potential reservoirs, and it has been suggested that Q fever can be sexually transmitted [34–36]. To this end, we assayed testes, adipose tissue, and bone marrow to determine if they could be sources of latent infections with these isolates. None of these tissue types had detectable levels of *C. burnetii* DNA at 12 weeks pi. While the limits of detection of our assays mean that we can’t discount them completely as reservoirs of latent infection, our data do not support these tissues as long-term reservoirs of *C. burnetii*.

In this mouse model, inflammatory cytokines were induced *in vivo* by ST16/26 and ST20 isolates. 9Mi infection typically induced the highest levels, but induction for most cytokines was observed with other ST16/26 and ST20 isolates. ST8 isolates and the AuQ7 and RT-Schperling isolates were very poor inducers of cytokines *in vivo*. These data show that robust *C. burnetii* infections can cause a broad cytokine response, but only a subset of sequence types is capable of generating detectable inductions. For many of the cytokines, greater sensitivity may be achieved by testing stimulated lymphocytes or PBMCs stimulated *in vitro* [24].

The antibody responses described show that while ST16/26 and ST20 isolates cause robust adaptive immune responses like those previously described, infection with ST8 isolates does not consistently stimulate detectable antibody responses during acute infection. The ST8 isolates caused Ph II antibody responses less than 50% of the time, and typically much later in the course of infection, and the ST8-infected animals that seroconverted were also the animals that had PCR positive tissues at 12 weeks pi. The PCR and serological data taken together could suggest that although ST8 isolates grow poorly in this *in vivo* mouse model, they are sometimes able to persist. This persistence could be enabled by their inability to induce robust immune responses.

While hemothoraxes have not been previously described in Coxiella infections, they are a known symptom of other bacterial pneumonias, such as those caused by tuberculosis infection. The finding of PCR positive hemothoraxes only in ST16/26- and ST20-infected mice could indicate that these sequence types are more likely to cause acute pneumonia upon aerosol infection. The fact that PCR positive hemothoraxes were not found in any mice infected with ST8 isolates, along with the reduced acute immune responses caused by these isolates, further indicates that ST8 isolates are less likely to cause symptomatic acute infections than ST16/26 and ST20 isolates.

Based on splenomegaly, weight loss, bacterial load, cytokine, and antibody responses, 9Mi had the most robust infection in mice compared to all other isolates. 9Mi-infected mice had the most severe clinical symptoms, with weight loss up to 20% (Figure 1) and higher levels of splenomegaly (Figure 2) relative to the other strains tested. All tissues tested from 9Mi-infected mice were PCR positive at 2 weeks pi, and most were still PCR positive at 12 weeks pi, unlike any of the other isolates used for infection. Furthermore, 9Mi-infected animals had antibody titers and cytokine levels much greater than those of any other infected group. The 9Mi isolate used for these studies (NM Phase I, clone 7) has a passage history that includes over 300 passages in guinea pigs. It is likely that 9Mi has become well adapted to infection in rodents and this may have led to its increased virulence in mice. Other studies comparing virulence of 9Mi to other strains have produced similar results [14,15,28]. These results suggest that 9Mi may be an outlier for animal infections, and other isolates may be more relevant as models for human infection.

This study was designed to assess the characteristics of contemporary sequence types of *C. burnetii* relative to one another, and to determine the relative virulence of these sequence types compared to 9Mi in a mouse model. The study has shown that 9Mi is the most virulent isolate that we studied, but other ST16/26 isolates also caused robust infections with somewhat lower splenomegaly, bacterial loads, and cytokine production. ST20 isolates also demonstrate virulence in this model and have overall results similar to the non-9Mi ST16/26 isolates. ST8 isolates had the lowest virulence. These isolates did not induce splenomegaly, weight loss, or cytokine induction. Low-titer antibody responses and low bacterial loads were only observed in some animals. The isolates tested that originated outside the U.S. showed mixed results. AuQ17 showed virulence like the ST8s, has the same plasmid and is closely related on the phylogenetic tree. RT-Schperling, which is genetically related to ST8 isolates but with a different plasmid, had virulence intermediate between ST8s and ST16/26. It induced cytokines poorly and induced low antibody titers but had reasonable bacterial loads at 12 weeks. Mice were followed to 12 weeks pi with the possibility of finding persistent *C. burnetii* in the tissues. Some *C. burnetii* DNA was observed in tissues at 12 weeks, but it remains to be determined if this represents slow clearance of *C. burnetii* DNA or persistent infection that could lead to chronic Q fever.

## Supplementary Material

Supplemental Material

## Data Availability

The datasets generated and analyzed during this study are available from the corresponding author upon request.
