# Supplementary figures and images for: Coxiella burnetii infections in mice: Immunological responses to contemporary genotypes found in the US
Source: Virulence. 2021 Sep 13;12(1):2461–73. doi: 10.1080/21505594.2021.1975527 (PMC8451504; doi:10.1080/21505594.2021.1975527)

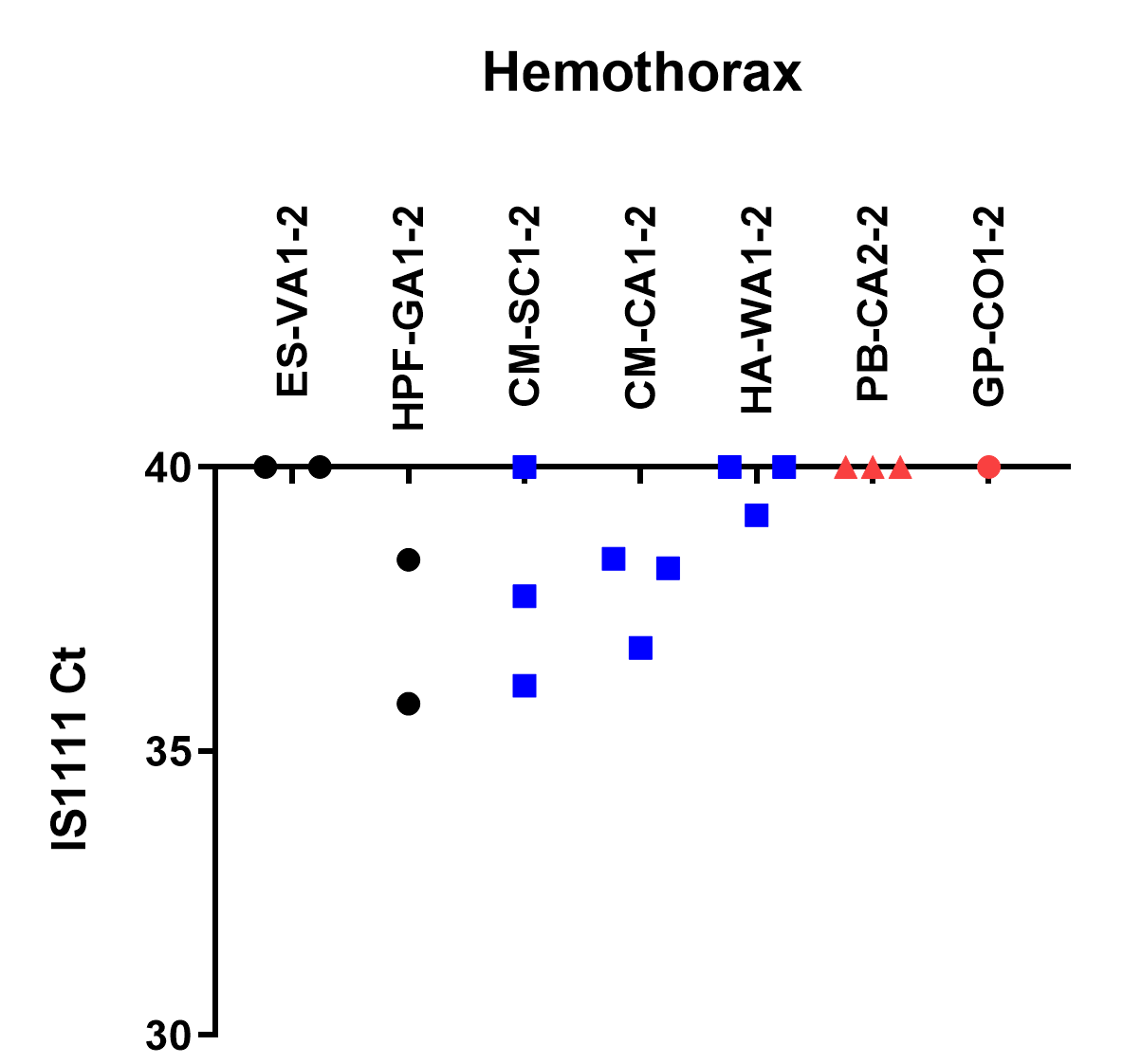

Supplement: Supplemental Material [file KVIR_A_1975527_SM2496.tif]
